# FACETS: multi-faceted functional decomposition of protein interaction networks

**DOI:** 10.1093/bioinformatics/bts469

**Published:** 2012-08-20

**Authors:** Boon-Siew Seah, Sourav S. Bhowmick, C. Forbes Dewey

**Affiliations:** ^1^School of Computer Engineering, Nanyang Technological University, Singapore, ^2^Biological Engineering Department, Massachusetts Institute of Technology, Cambridge, MA 02139, USA and ^3^Singapore-MIT Alliance, Nanyang Technological University, Singapore

## Abstract

**Motivation:** The availability of large-scale curated protein interaction datasets has given rise to the opportunity to investigate higher level organization and modularity within the protein–protein interaction (PPI) network using graph theoretic analysis. Despite the recent progress, systems level analysis of high-throughput PPIs remains a daunting task because of the amount of data they present. In this article, we propose a novel PPI network decomposition algorithm called FACETS in order to make sense of the deluge of interaction data using Gene Ontology (GO) annotations. FACETS finds not just a single functional decomposition of the PPI network, but a *multi-faceted atlas* of functional decompositions that portray alternative perspectives of the functional landscape of the underlying PPI network. Each *facet* in the atlas represents a distinct interpretation of how the network can be functionally decomposed and organized. Our algorithm maximizes interpretative value of the atlas by optimizing *inter-facet orthogonality* and *intra-facet cluster modularity*.

**Results:** We tested our algorithm on the global networks from *IntAct*, and compared it with gold standard datasets from MIPS and KEGG. We demonstrated the performance of FACETS. We also performed a case study that illustrates the utility of our approach.

**Contact:** seah0097@ntu.edu.sg or assourav@ntu.edu.sg

**Supplementary information:** Supplementary data are available at the *Bioinformatics* online.

**Availability:** Our software is available freely for non-commercial purposes from: http://www.cais.ntu.edu.sg/∼assourav/Facets/

## 1 INTRODUCTION

The massive amount of biological interaction datasets presents the opportunity to study higher order organization and modularity of interaction networks. High-throughput interaction experiments, however, introduce new challenges to visualization and analysis of biological interaction data. A common thread that runs through high throughput generated data is information overload, i.e. the explosion of data that makes intuitive and meaningful functional analysis difficult, even overwhelming. In case of protein–protein interaction (PPI) data, decomposing the network into functional modules is often the key step to understanding the overall picture of the functional relationships that underlie the data. Consequently, graph clustering methods that decompose PPI networks into their functional constituents are increasingly pertinent (Lavallée-Adam *et al.*, 2009).

**Fig. 1.**
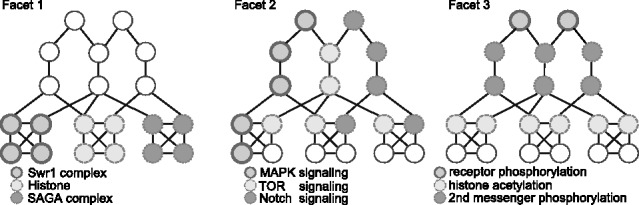
Illustration of multi-faceted PPI network decomposition

In general, graph clustering algorithms discover regions of dense connectivity that represent protein complexes or functionally coherent processes (Bader and Hogue, 2003; Krogan *et al.*, 2006; Seah *et al.*, 2012). Unfortunately, these methods output *only a single optimal functional decomposition* of the PPI network. Consequently, a PPI network can only be decomposed and viewed from a single perspective, whereas in reality there are often multiple different perspectives (decompositions) associated with the functional organization of the underlying network, all of which are distinct and equally valid. We refer to each of these decompositions as a *facet* because they visualize the organization of a PPI network from a unique view, providing a distinct interpretation of the organization of the underlying network. For example, consider the toy transcriptional regulatory network depicted in Figure 1. A typical decomposition, based on an existing graph clustering technique (e.g. mcode in Bader and Hogue, 2003), identifies dense regions of the network, which correspond to the decomposition of protein complexes as shown in *Facet 1*. However, this network can also be viewed from other different perspectives. For instance, it can be organized by the types of signaling pathways involved in it (*Facet 2*). Notice that the decomposition from this perspective is markedly different from the complex-based decomposition. Furthermore, different proteins in the network may undergo various modifications such as acetylation, phosphorylation and ubiquitination. Hence, yet another way to decompose the network is by their modification effects as depicted in *Facet 3*. Clearly, in larger real-world networks the possibility of uncovering multiple, distinct functional decompositions are real.

At first glance, it may seem that we can tune the clustering parameters of existing graph clustering techniques in order to generate multiple facets or decompositions. Unfortunately, such tuning only generates an exponential number of *slightly perturbed* decompositions with incremental differences (see Supplementary Material). In other words, such strategy does not generate functionally unique decompositions. In contrast, it is imperative to ensure that the decompositions or facets are *distinctive*, i.e. they are maximally different from each other. This is because every facet should provide a fresh and informative perspective to the organization of the network, rather than providing just incremental differences with respect to other facets.

*Our contribution.* We propose a novel algorithm called FACETS that discovers an *atlas* of functionally unique decompositions from a PPI network, portraying alternative views of the functional landscape of the network (detailed in Section 2). Each decomposition or facet represents a distinct interpretation of how the network can be functionally decomposed and organized. Since a key objective is to obtain *n* unique facets that are informative and orthogonal (We use the term orthogonal to describe the idea of distinctive clusters, rather than its precise mathematical meaning.), our algorithm maximizes interpretative value of the atlas by optimizing *intra-facet cluster modularity* and *inter-facet orthogonality*. *Intra-facet cluster modularity* captures the aim of decomposing a PPI network *G* based on a particular functional and/or structural view. For instance, based on complexes and localized structures, *G* can be decomposed into protein complexes. If we consider regulatory processes as a functional concept, then *G* can be decomposed into signaling and regulation pathways, an entirely different decomposition. *Inter-facet orthogonality*, on the other hand, demands that each of the *n* facets are structurally distinctive and functionally apart from each other. We propose a novel *objective function* that models these intuitions and FACETS exploit it to discover a set of distinct facets. Specifically, we exploit both the PPI graph structure and the rich functional information provided by Gene Ontology (GO) annotations to guide facets construction.

In Section 3 we evaluate the performance of FACETS on real world PPI datasets. We also compare FACETS-generated decompositions against several gold standard datasets. We demonstrate its superiority over tested graph clustering methods. We illustrate the robustness of FACETS against noise. Finally, we conduct a case study to illustrate how multi-faceted decompositions identified multiple organization maps of the human autophagy system (Behrends *et al.*, 2010).

*Related work.* Multi-view clustering is a poorly studied problem in the data mining community (Qi and Davidson, 2009). Still, there are several works that have focused on multi-view clusterings in image and text mining domain (Niu and Dy, 2010). One approach projects data into an alternative subspace (Cui *et al.*, 2007). Another approach generates alternative clustering through the use of *must-link* and *cannot-link* constraints (Wagstaff and Cardie, 2000). In meta-clustering (Caruana *et al.*, 2006), a large number of clusters are generated and clusters which are truly different are selected. All of the aforementioned approaches, however, assume data points in the *vector space* that allow the notion of metric distances in a Euclidean geometry. On the other hand, our problem demands a multi-view clustering methodology on *attributed graphs*, which requires a graph clustering paradigm on both structure and annotation. To the best of our knowledge, multi-view clustering paradigm has not been applied in clustering biological networks to identify pertinent functional modules from multiple perspectives.

Ensemble clustering methods generate an ensemble of near-optimal decompositions (Agarwal and Kempe, 2008; Massen and Doye, 2005; Navlakha and Kingsford, 2010). These methods have been used to increase the quality and confidence of the decomposition and understand network dynamics. The near-optimal decompositions generated, however, have no notion of the orthogonality that this work is seeking. Instead, ensemble clusterings create a large number of perturbed solutions, making them unsuitable as an atlas of functionally distinct decompositions. For instance, in (Navlakha and Kingsford, 2010), a small network of 32 nodes generated at least 82 permutations of clusterings.

## 2 MATERIALS AND METHODS

In this section, we formally introduce the multi-faceted functional decomposition problem. We begin by defining some terminology that we shall be using in the sequel. We use the network in Figure 1 as running example in this article.

### 2.1 Terminology

An undirected network *G* = (*V, E* ) contains a set of vertices *V*, representing biological entities like proteins or genes, a set of edges *E*, representing interactions between the entities. A *functional module* 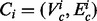 is a subnetwork of *G* such that 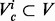 and 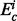 is the set of edges induced by 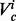 from *G*. A *facet* (*decomposition* or *view*) of *G*, denoted by *F*, is a set of functional modules 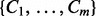 representing a specific functional concept. Functional modules within a facet *F* are allowed to overlap. In the sequel, we use the terms facet, view and decomposition interchangeably. A *functional atlas* (or atlas for brevity) of *G*, denoted by *A*, is a set of facets 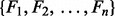 that represents distinctive functional landscapes of *G*. Figure 1 depicts an atlas of three facets, with each facet decomposing the network into three functional modules.

In order to support the idea of functionally orthogonal views, we utilize GO annotations associated with proteins. Given a GO directed acyclic graph (DAG) 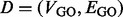, the ordered set 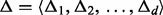 is a topological sort of *D*, where 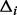 represents a single GO term. Each vertex 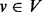 is associated with a *d*-dimensional *function association vector* 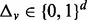, such that 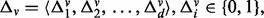 where 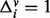 if and only if the term 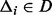 or its descendants are associated with protein *v*, and 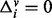 if otherwise. Note that 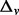 is an indicator vector that indicates GO terms that are associated with *v*.

A *facet candidate bundle* 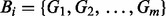 is a set of connected subnetworks of *G* such that for every 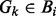, there is a shared GO term 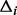 within every 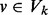. 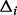 represents the common function of the candidate subnetwork. A facet candidate bundle 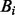 represents the superset of facet 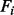 and it contains a large permutation of subnetworks that satisfy a particular functional concept. Typically, 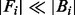. A *function bundle* 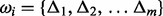 is the set of shared GO annotations of 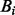, i.e*.* 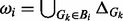. To illustrate these concepts, consider the PPI network in Figure 1. Suppose that 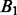 is a facet candidate bundle with 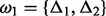, where 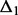 represents the Swr1 complex GO term and 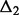 the Histone term. In the subgraph with Swr1 complex label in Facet 1, every node in that subgraph is annotated with Swr1 complex term. Thus, the subgraph is a valid member of 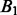. Any subgraph made up of Histone-labeled nodes is also a valid member of 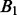. If 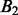 represents the facet candidate bundle with 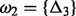, where 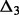 represents cellular component, then the Swr1 complex-labeled subgraph is also a valid member of 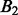 (Swr1 complex is a cellular component). Furthermore, every subgraph in Facet 1 whose nodes are labeled is a valid member of 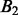, but not neccessarily a valid member of 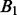. One can see that 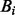 contains a set of subgraphs that shares specific functional concepts depending on the functional terms in 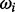. We define the function *f* : *P* (*V*_*go*_) → A given by 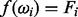 to make explicit the association between a functional bundle and its corresponding facet.

A *function bundle partition* 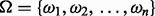 is the set of function bundles that form a partition of all GO terms 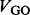, i.e*.* 
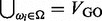
. In the next section, we shall impose further constraints on facet candidate bundles and function bundles such that the shared GO terms of the subnetworks within each facet candidate bundle shares high functional commonality and the terms shares in one facet are distinct from the terms in another facet.

### 2.2 Problem formulation

The goal of multi-faceted functional decomposition problem is to identify an atlas of *n* distinct facets of *G* that maximizes *inter-facet orthogonality* and *intra-facet cluster modularity*. Each facet depicts a higher-order organization of modules of *G*. Recall that inter-facet functional orthogonality demands that each of the *n* facets is based on an orthogonal functional concept—facets that are distinctive and functionally apart from each other. Hence, we propose two criteria that model the intra-facet functional modularity and inter-facet orthogonality of an atlas solution. Next, we propose an *objective function* that models and scores an atlas of *G*.

#### 2.2.1 Intra-facet cluster modularity

Intra-facet cluster modularity enables us to seek clusters that are both structurally and functionally modular. Given 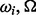 and *G*, 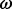
*-restricted* decomposition procedure (denoted by 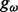) computes a decomposition of *G* into 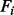 such that 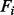 satisfies the following criteria:

*Criterion 1.* Every module 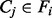 should be *functionally bounded* by 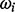. Let 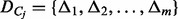 be the set of shared terms in 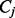, i.e*.* for every 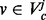, *v* must be annotated with every 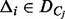. Then, the *functional boundedness* of module 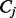 by 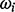 is given by 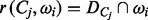. A cluster 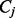 is bounded by 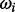 if 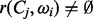. An 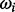-restricted decomposition of a facet draws from a restricted search space of subnetworks in *G* whose vertice shares at least a term within 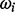. Intuitively, this means that for any subnetwork to be considered as a module, it must first be sharing a term in 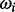. Even if a subnetwork is dense, it must yield to sparser subnetwork candidates if it is not enriched with terms within 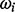. In the example of Figure 1, if 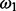 is terms of protein complexes, then any subgraphs enriched with complex terms is in the search space for *Facet 1*. In contrast, the modules of *Facet 2*, enriched with signaling terms, would be invalid candidates for *Facet 1* decomposition. This restricted search space is modeled by facet bundle 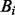, where any valid candidate facet cluster 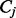 of facet 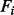 must belong to 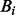.

*Criterion 2.* A facet 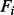 decomposes *G* by maximizing a clustering objective function 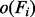 while satisfying the above criterion. These criteria are determined by the specific graph clustering algorithm that is adapted for creating a facet; for generality we let this be the objective function 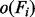 that has to be maximized by the graph clustering algorithm. For instance, every module 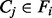 has to be structurally dense and/or functionally coherent (i.e*.* every node in module shares a common function), the coverage of 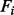 has to be high, and the amount of overlap between modules should be low. For example, modules of *Facet 2* maximize 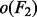 while satisfying the 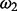 bound, despite not forming dense modules. This is because all dense modules formed are enriched with complex terms, violating the 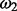 bound.

#### 2.2.2 Inter-facet orthogonality

Since we want every facet in the atlas to be functionally and structurally distinct, modules within a facet, as whole, should be structurally and functionally distinct from modules within another facet. We discuss two independent distance measure between facets: *functional orthogonality* and *structural orthogonality*.

*Functional orthogonality* is indirectly controllable by the function bundles attached to facets, which determines the types of allowable modules through the aforementioned restriction. By increasing inter-bundle functional orthogonality, we increase the functional distinctiveness of each facet. To impose functional orthogonality, we introduce the following constraint: for every 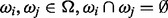 and 
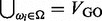
. This requires that 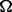 actually partitions the terms of the GO DAG. The *functional distance measure* between 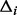 and 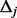, denoted by 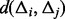, measures the functional dissimilarity between the terms. In this article, 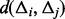 is simply computed as the length of the shortest path between the terms: 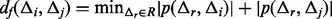, where *R* is the set of common ancestors of term 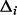 and 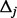 and 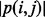 is the length of the shortest path from node 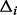 to 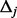 in *D*. The *candidate function specificity* 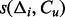 is defined as 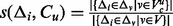. 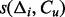 measures the specificity of a shared GO term, which we will later use to weigh the contribution of the term. For instance, a cluster 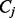 of 5 nodes that share the biological process GO term in a network of 1000 biological process annotated nodes has a low specificity value of 0.005 with respect to the term.

Likewise, we define structural orthogonality. The *structural distance measure* between two clusters 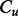 and 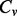 is defined as 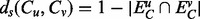 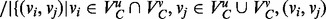 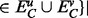. The distance is 0 if 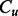 and 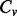 shares all edges and 1 if 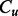 and 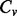 shares no edges.

Following that, we define 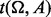 as the linear combination of inter-facet functional and structural orthogonality, as follows:

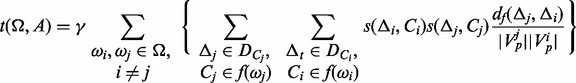


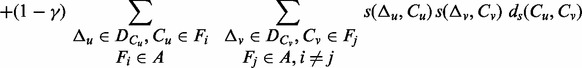



The parameter 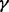 weighs the contribution of 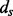 against 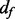, and is set to attain balanced contribution from both terms. Note that 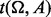 quantifies the pairwise orthogonality between two function bundles. The higher the score, the greater the orthogonality.

### 2.3 Problem definition

The multi-faceted functional decomposition of *G* is defined as the problem of simultaneously constructing the atlas of decompositions 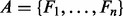, and the function partition 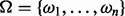, such that the following objective function is maximized:

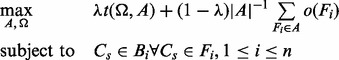



The right half of the terms captures the cost function of decomposing *G* into *A*; the left half, decomposing *D* into 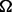. The parameter 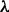 controls the weightage between the two terms. Observe that one has to optimize these criteria simultaneously over the space of *A* and 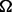. Otherwise, one may end up with a poor objective score. For instance, if 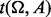 is high (meaning highly orthogonal partitioning), but 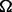 is improperly partitioned such that one ends up with 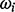 that allow only poor decompositions, then the 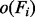 score would be very low. Due to the interdependence of the criteria, optimizing the aforementioned function is computationally expensive.

### 2.4 FACETS algorithm

**Fig. 2.**
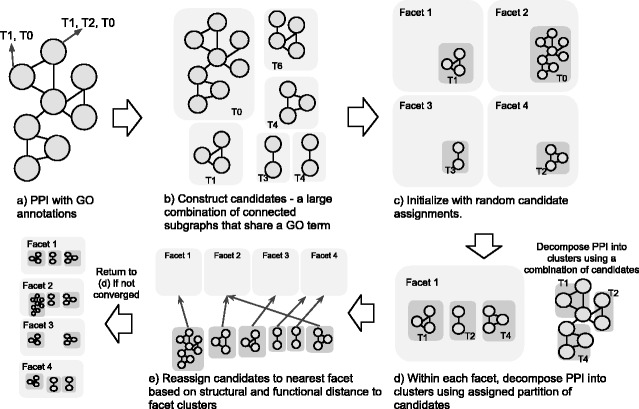
Illustration of the FACETS algorithm. (**a**) GO annotated PPI network is used as input. (**b**) The set of candidate subnetworks are computed. (**c**) An initial set of modules are randomly assigned to a facet. Candidate subnetworks are then assigned to their nearest facet based on function and structure distance. (**d**) For each facet, decomposition is performed to identify modules that are functionally contained by the facet candidate bundle. (**e**) The candidate subnetworks are reassigned based on their distance to the new set of modules identified. Convergence is achieved when the number of terms reassigned to a different facet drops below the threshold parameter θ. Otherwise, Steps (d–e) are repeated

Generally, the problem of finding clusters that maximizes typical clustering objective functions that relate to graph density is known to be NP-hard (Jagota, 1995). Hence, the FACETS algorithm is a heuristic implementation that attempts to find a local maximum of the objective function. Our heuristics is a step-wise iterative approach that incrementally optimizes 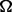 and *A*, one at a time (Fig. 2). Intuitively, given an attributed PPI network (e.g*.* Fig. 2a), 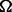 is incrementally updated by using each facet in *A* as functional centroids, and then using the centroids to partition *D*. *A* is updated through 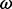-restricted decomposition using the updated 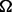. The FACETS algorithm consists of two phases: the *initialization* phase (Fig. 2b), and the *iteration* phase (Fig. 2c–d) (see Supplementary Material). We describe each of them in turn.

*Initialization*. This phase creates an initial set of decompositions for the second phase. We perform graph clustering on *G* to obtain an initial set of modules. To this end, the FUSE (Seah *et al.*, 2012) algorithm, summarized in Supplementary Material, is utilized. Each module of this set is then randomly associated with a facet, randomly distributing the modules over an initial set of facets. Following that, we construct *candidates subnetworks*, which use subnetworks of *G* that satisfy 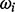-restricted decomposition constraint. To generate candidates exhaustively is prohibitively expensive. Instead, candidates for a facet 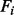 are generated as follows: for every GO term 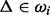, we obtain the induced subnetwork in *G* whose nodes are annotated with 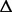 or its descendants. The subnetwork is then decomposed into connected components, each forming a candidate subnetwork 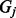. Let 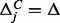 be the term associated with this candidate. Candidates formed this way can vary greatly in resolution of the annotation that its nodes share (for example, 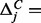 biological process), and can be highly overlapping.

*Iteration.* This phase—the actual optimization phase—is performed in rounds. Let *i* denote the *i*-th iteration of the algorithm. At each round, the algorithm updates *A* and 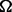 in two sequential steps. To evaluate algorithm convergence, we introduce *functional reassignment—*the number of terms in 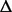 that is reassigned to a different function bundle after Step 1 of *i*-th iteration. This score measures the rate of change of 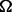, indicating how close the algorithm is to convergence. Observe that when 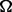 is fixed, the algorithm reaches a steady state. The algorithm reaches convergence and terminates when the functional reassignment at *i*-th iteration drops below *convergence threshold* 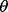, a user-defined parameter.

*Step 1.*** Update** 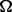. In this step, we assume that *A* is a constant and update 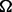 to increase 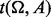. For each 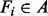, the enriched functional terms of the modules in 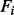 serve as centroids for partitioning *D* into orthogonal concepts; these enriched terms as whole form the centroid of 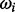, which is associated with 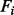. We then reassign every candidate subnetwork to its nearest centroid to form a partition 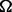. The convergence properties of such centroid-based partitioning approaches (e.g*.* K-means) has been well studied (Bottou and Bengio, 1994). For every 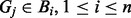, we determine its *closest* centroid by considering 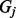
’s average functional and structural distance to functional modules within a facet. The facet that is closest to 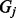 is indicated by:

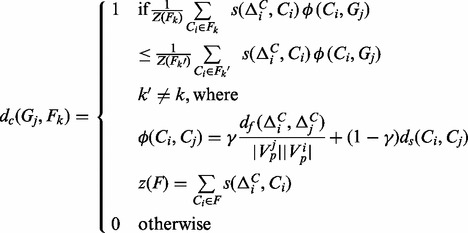



Following that, 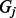 is reassigned to nearest facet candidate bundle 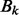 (superset of 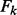) and 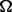 is updated based on where every 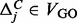 is assigned to. Each function bundle 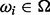 represents functional terms that are most closely associated with 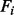, and the decomposition of 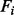 in the following step will be bounded by the updated 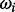. Function partitioning depends on the atlas of decompositions because not every partition of the GO DAG is capable of forming a modular decomposition of functional modules.

*Step 2***. Update *A***. In this step, we update *A* to maximize the objective function while fixing 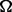. To support 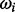-restricted decomposition of 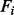, we propose an algorithm that employs profit maximization model (discussed below) and runs in iterations. At each iteration, we score candidate subnetworks based on a profit maximization model and greedily selects the best scoring candidate as member in 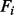. An iteration runs for every 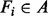 before moving to the next iteration. Every candidate considered for 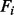 must satisfy the 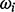-restricted decomposition constraint, i.e*.* the candidate subnetwork must be enriched with terms in 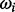. In other words, 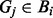.

We now describe the profit maximization model for scoring a candidate 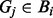. Every 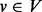 is assigned an information budget. A candidate 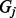 extracts, from each 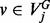, some information revenue from the budget pool. The revenue extracted is correlated to the edge density of the subnetwork, with modular candidates giving high revenue. Each time a candidate is selected, revenue is removed from the budget pool and a cost is incurred. A penalty cost is incurred for a candidate that is structurally similar to selected clusters in other facets 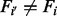. This penalty is modeled by 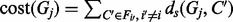, which utilizes the structural distance measure 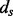 described earlier. At each iteration, the candidate that contributes the highest information profit (revenue minus cost) is selected. To summarize, a clustering in 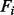 that yields high overall revenue have subgraphs with high facet modularity 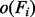, whereas a clustering with low overall cost yields high inter-facet orthogonality 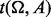. Given a fixed 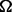, the set of facets *A* with maximum overall profit maximizes the objective function. The algorithm above approximates this through greedy heuristic.

## 3 RESULTS

### 3.1 Experiment settings

The FACETS algorithm is implemented in Scala (Odersky *et al.*, 2004). We now present the experiments conducted to study the performance of FACETS and report some of the results here. All experiments were executed on a 1.66 GHz Intel Core 2 Duo T5450 machine with 3GB memory. We primarily used the global human PPI network from *IntAct* (Kerrien *et al.*, 2007), as well as the *yeast*, *fruit fly*, and *human autophagy* networks from *IntAct* (Table 1). In all experiments, we set the convergence threshold 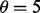. The weight 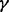 is set to 0.091 to balance the contribution of structure and function (equal order of magnitude). We utilize only the cellular process sub-domain of the GO so that the facets are created not merely based on different GO domains, but on more subtle functional differences.

**Table 1.** Datasets used

Dataset	No of nodes	No of edges	Source
*H. sapiens*	9131	34 362	IntAct (Kerrien *et al.*, 2007)
*S. cerevisiae*	4768	40 457	IntAct
*D. melanogaster*	3114	6472	IntAct
Human autophagy	1241	3555	IntAct

#### 3.1.1 Evaluation criteria

To measure the similarity/dissimilarity between facets or decompositions, we employed the *Jaccard index* (JI) (Ben-Hur *et al.*, 2002) evaluation measure, which is widely used to compare clusterings based on counting the agreement or disagreement of co-clustered pairs of proteins. The reader may refer to Supplementary Material for definitions of the measure.

### 3.2 Experiment results

#### 3.2.1 Quantitative assessment

Table 2 shows the quantitative comparison between facets. We measure the inter-facet decomposition similarity using the JI score. The low clustering similarity scores between facets show that they are decomposed distinctively. This reflects significant organizational differences between modules of signaling pathways and modules of protein complexes. We measured the *coverage* of a facet and the *extent* of coverage overlap between the facets. Let the coverage of a facet *F_k_* be 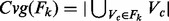. Also, let the extent of coverage overlap between the *F_i_* and *F_j_* be 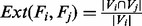, where 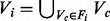 and 
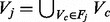
. The extent of overlap between facets reaches up to 0.316. Consequently, the overlap is not insignificant, implying that the facets are not partitions of *G*.

**Table 2.** Comparison between facets of the *H. sapiens* PPI network (*n* = 6)

			JI score						Coverage overlap					
Facets	No of modules	Coverage	Facet 1	Facet2	Facet 3	Facet 4	Facet 5	Facet 6	Facet 1	Facet 2	Facet 3	Facet 4	Facet 5	Facet 6
1	89	294	1.0	0.014	0.065	0.0050	0.0070	0.079	1.0	0.316	0.142	0.081	0.044	0.112
2	280	1079	0.014	1.0	0.0040	0.119	0.0050	0.0070	0.086	1.0	0.077	0.09	0.082	0.079
3	106	372	0.065	0.0040	1.0	0.0010	0.0	0.013	0.112	0.225	1.0	0.029	0.059	0.086
4	94	419	0.0050	0.119	0.0010	1.0	0.0	0.0080	0.057	0.233	0.026	1.0	0.028	0.052
5	114	390	0.0070	0.0050	0.0	0.0	1.0	0.0010	0.033	0.228	0.056	0.03	1.0	0.038
6	72	306	0.079	0.0070	0.013	0.0080	0.0010	1.0	0.107	0.281	0.104	0.071	0.049	1.0

#### 3.2.2 Validation on real data

**Fig. 3.**
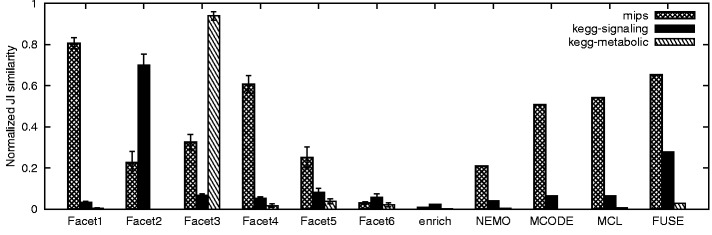
Comparison between the decomposition similarities of FACETS, other methods and gold standard decompositions

In this experiment, we compare the FACETS atlases of the global human network to gold standard functional modules. The gold standard datasets were constructed as follows: (i) MIPS—We use the set of 571 human complexes (of more than three proteins) from MIPS (Mewes *et al.*, 2002) to represent the decomposition of the human interactome into complexes. (ii) KEGG-metabolic—To represent decomposition into metabolic modules, we use 67 human metabolic networks from KEGG, each representing a single functional module. (iii) KEGG-signaling—We use 23 human signal transduction pathways from KEGG to represent decomposition into signaling pathways. The gold standard decompositions were chosen such that each represents a distinct functional organization of the human network. As such, we consider each gold standard dataset as a facet of the human network, and the set of these three datasets as the gold standard atlas of the human network. We then compared these datasets against the atlas of facets obtained through our algorithm and determine if there is a distinctive one-to-one mapping between our facet and a gold standard facet. We set *n* = 6 and repeated the tests fifteen times under different starting conditions to account for variability in facets output. We also compare the similarity scores against graph clustering methods, namely Markov clustering (MCL) (Krogan *et al.*, 2006), mcode (Bader and Hogue, 2003), nemo (Rivera *et al.*, 2010) and fuse (Seah *et al.*, 2012). These methods create a single decomposition of the human network. We removed clusters with fewer than three proteins. We also compare against GO term enrichment (enrich) (Boyle *et al.*, 2004), which does not utilize structural information. Following that, we measure the clustering similarities between the gold standard datasets and the decompositions obtained. Figure 3 shows the clustering similarities between modules in gold standard datasets and modules in facets as well as tested graph clustering methods. The JI was used to measure the agreement between pairs of decompositions. We normalize the scores so that the highest JI score obtained, within each gold standard dataset, is adjusted to 1.

We consider the facet best associated with a gold standard decomposition by comparing their relative scores. The gold standard datasets were uniquely mapped to a distinct facet: KEGG-metabolic is most similar to *Facet 3*, KEGG-signaling is most similar to *Facet 2* and MIPS is most similar to *Facet 1*. This unique mapping demonstrates that from a clustering perspective, the facets have significant functional orthogonality such that they are uniquely associated with different functional organization maps. *Facet 6* has poor similarity to the gold datasets, indicating a set of clusters that could be functionally distinct from these datasets.

In contrast, the tested graph clustering methods share common similarity patterns. Clusters are largely from a single dominant perspective—those of protein complexes (MIPS). We argue that objective functions based on dense connectivity tend to favor protein complex structures over other decompositions like metabolic pathways. GO term enrichment, on the other hand, generates output with little similarity to all gold standard datasets, indicating that annotations alone are unable to specifically identify important functional modules within a large PPI network. This is supported by the fact that functional analysis of large networks often involve graph clustering prior to term enrichment (Krogan *et al.*, 2006).

#### 3.2.3 Robustness

**Fig. 4.**
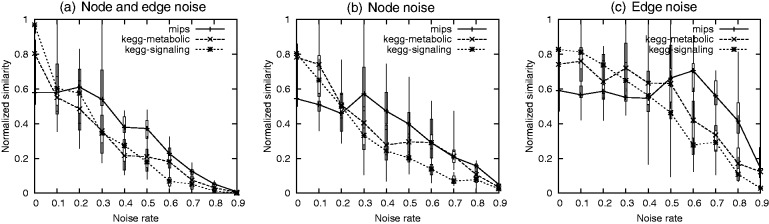
Effect of noise on FACETS algorithm

To study the robustness of FACETS, we tested the effect of annotation perturbations and edge deletions of the input network on FACETS output. Random edge deletion (*edge noise*) simulates the effect of removing false positive interactions in high-throughput interaction datasets, whereas annotation perturbation (*node noise*) simulates errors in curated annotations. Figure 4(a–c) shows the effect of edge and node noise on FACETS, varying from 0 to 100% noise. The figure shows clustering similarities (JI similarity) between the best scoring facets and gold standard datasets under increasing noise perturbations. We repeated each test fifteen times with different randomization seed. We observed that FACETS output quality drops gradually under increasing edge and node noise conditions. This demonstrates that the algorithm is robust to small noise perturbations. In case of edge noise, we noted that the quality of output only drops rapidly past the 0.5 noise ratio. This is desirable given that false-positive rates in yeast two-hybrid and TAP experiments range between 0.35 to 0.7 (Hart *et al.*, 2006). MIPS clusters, which consist of densely interconnected clusters, are most robust to edge noise effects. The effect of node noise is comparatively greater, but quality degradation remains gradual.

#### 3.2.4 Effect of initial starting point

**Fig. 5.**
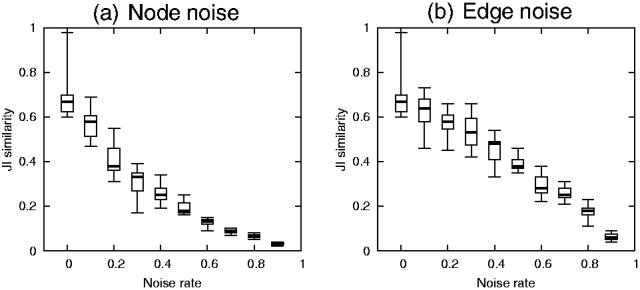
Effect of initial starting point versus noise on FACETS algorithm

Given that FACETS belong to the class of hill-climbing methods, the algorithm output is dependent on the initial starting point. To this end, we study the effects of multiple random initial starting points. We compared the variability in clustering output due to starting point versus variability due to noise effects to give a sense of the magnitude of variability. We set a single facet output as the reference output, and compared its JI similarity with outputs from different starting points and increasing noise effects. The boxplot Figure 5(a and b) shows the effect of initial starting point versus noise on facets. At 0 noise rate, the variability in JI similarity is due to initial starting point. Given the fact that high-throughput datasets are inherently noisy (as mentioned above), the variability due to starting point is less significant. In addition, Figure 4(a–c) shows the effect of starting points with respect to gold standard datasets when one observes the similarity at 0 noise rate.

#### 3.2.5 Convergence

**Fig. 6.**
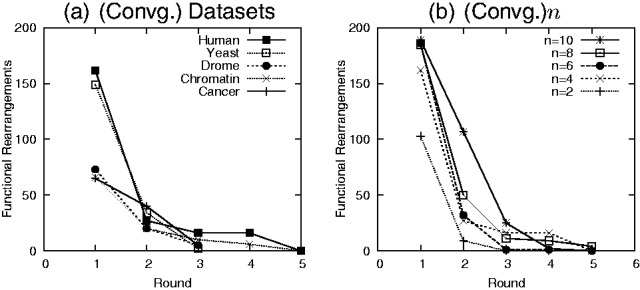
Rate of convergence of FACETS algorithm

Figure 6(a and b) shows the functional reassignments after the *i*-th iteration. We conducted the tests on varying types of datasets with *n* = 6. We also vary the number of facets per atlas (*n* = 2–6) on the global human network. All tests converge in <9 rounds, demonstrating FACETS’ ability to converge quickly to a solution. Larger datasets such as the human network requires more iterations to complete. The number of iterations required also tends to increase with the number of facets *n*.

#### 3.2.6 Case study: human autophagy system

**Fig. 7.**
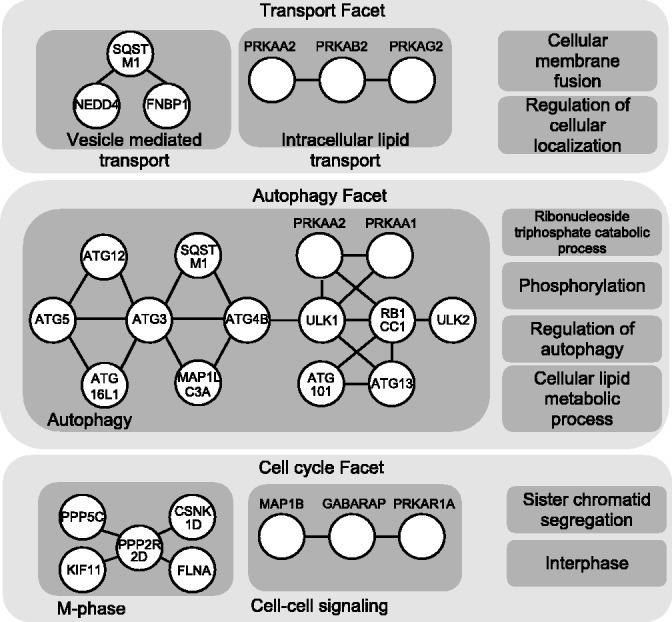
Multiple facets (subset) illustrating the functional organization of the human autophagy network under different perspectives

To illustrate the utility of multi-faceted decomposition, we analyze the functional organization human autophagy system. The functional map of this system was manually constructed in (Behrends *et al.*, 2010). We generated the facets of the human autophagy network (*n* = 6), and a subset of the results is shown in Figure 7. The automatically generated facets show the pertinent roles of vesicle transport and lipid membrane metabolism in autophagy, which is consistent with the manually constructed map. Additionally, the network can also be clustered from the perspective of cell cycle and apoptosis regulation modules, which is not depicted in the manual map. This demonstrates the possibility of having multiple perspectives that organize a network.

## 4 CONCLUSION

In this article, we propose FACETS, a data-driven and generic algorithm for generating multi-faceted functional decompositions of a PPI network, providing multiple perspectives of the functional organization landscape of the network. Our experimental validation with real-world PPI networks demonstrates effectiveness of FACETS in generating functionally distinctive facets. As future work, we intend to extend FACETS to evaluate both annotated and unannotated regions of the PPI network.

*Conflict of Interest*: none declared.

## Supplementary Material

Supplementary Data
