# Supplementary material for: FACETS: multi-faceted functional decomposition of protein interaction networks
Source: Bioinformatics. 2012 Aug 20;28(20):2624–31. doi: 10.1093/bioinformatics/bts469 (PMC3467740; doi:10.1093/bioinformatics/bts469)
Supplement: Supplementary Data [file supp_28_20_2624__index.html]

FACETS: multi-faceted functional decomposition of protein interaction networks — Supplementary Data 

# FACETS: multi-faceted functional decomposition of protein interaction networks

## Supplementary Data

files

**Files in this Data Supplement:**

- Supplementary Data - pdf file
