# Supplementary material for: FACETS: multi-faceted functional decomposition of protein interaction networks
Source: Bioinformatics. 2012 Aug 20;28(20):2624–31. doi: 10.1093/bioinformatics/bts469 (PMC3467740; doi:10.1093/bioinformatics/bts469)
Supplement: Supplementary Data [file supp_bts469_facets-bioinformatics_supplementary.pdf]

1 SUPPLEMENTARY MATERIAL A: THE FUSE ALGORITHM.

The FUSE algorithm finds a k-module decomposition of a PPI network. It is based on a profit maximization principle, which posits that networks contain a limited budget of information and that modules from these networks have varying level of informativeness. The most informative set of modules are then extracted from the pool of information budget using a greedy approach (information extracted are not returned to the pool). The FUSE algorithm ranks informativeness of a module based on its structural information content, with dense regions significantly more informative than random clusters. A size constraint cost is also introduced as regularization parameter to prevent imbalanced sized modules. The concept of limited budget constraint also models the diminishing returns of capturing the same proteins under multiple modules. This in turn captures the cost of including highly overlapping modules in the profit maximization model. The profit maximization problem is modeled as a variation of the budgeted maximum coverage problem, which explains the choice for using a greedy heuristics approach. The algorithm first assigns every protein in the network with a constant information budget. Candidate modules are then generated from GO terms, where each module contain proteins with a common GO term. The greedy algorithm that selects the set of k most informative modules that as a whole best summarizes the original network. Every time a module is selected, information profit (based on informativeness minus cost) is extracted from the remaining budget of affected proteins.

2 SUPPLEMENTARY MATERIAL B: PSEUDOCODE OF FACETS

Algorithm 1 FACETS

Input: G, D, n, θ, b **Output:** n facets $A = \{F_1, \ldots, F_n\}$

$F_0 \leftarrow$ Decompose G.
Initialize $A = \{F_1, \ldots, F_n\}$ by randomly distributing $C \in F_0$.
$r \leftarrow \infty$.
while $r > \theta$ **do**
  $\Omega \leftarrow \{\omega_1, \ldots, \omega_n\}$ where $\omega_i = \emptyset$
  for $G_i \in B_k, k = 1, 2, \ldots, n$ **do**
    Reassign G_i to nearest B_k
  end for
  Recompute ω_i from B_i and Update Ω
  $A \leftarrow \{F_1, \ldots, F_n\}$ where $F_i = \emptyset$
  Set for each $v \in V, F_i \in A$, $BudgetMap_{F_i}(v) \leftarrow b$
  while $profit > 0$ **do**
    $profit \leftarrow 0$
    for $F_i \in A$ **do**
      $profit, F_i, BudgetMap_{F_i} \leftarrow$
      OmegaDecompose($profit, BudgetMap_{F_i}, F_i, \Omega, A, G, b$)
    end for
  end while
  $r \leftarrow |\{\Delta_j : \Delta_j$ reassigned to a different facet, $\Delta_j \in \Delta\}|$
end while
return A

Algorithm 2 OMEGADECOMPOSE

Input: $profit$, $BudgetMap$, F_i, Ω, G, A, b **Output:** $profit, F_i, BudgetMap$

$Candidates \leftarrow \emptyset$.
$\omega_i \leftarrow \omega_i \in \Omega$ associated with F_i.
for $\Delta \in \omega_i$ **do**
  $G_\Delta \leftarrow$ induced subgraph of G that shares Δ annotation or its descendents
  $C_\Delta \leftarrow \{g|$ connected components of $G_\Delta\}$
  $Candidates \leftarrow Candidates \cup C_\Delta$
end for
for $C \in Candidates$ **do**
  $revenue(C) \leftarrow o(C)$, where $o(C)$ is the clustering objective score of C taken from $BudgetMap$
  $cost(C) \leftarrow d_c(C)$ given A
  $profit(C) \leftarrow revenue(C) - cost(C)$
end for
$F_i \leftarrow F_i \cup \{C|$ candidate with max $profit(C)\}$
$profit \leftarrow profit+$candidate with max $profit(C)$
Update $BudgetMap$ based on candidate with max $profit(C)$
return $F_i, profit, BudgetMap$

Theorem 1 Algorithm FACETS takes $O(n|\Delta||V| + n|\Delta|^2|V|^2)$ time per iteration in the worst case.

PROOF. In step 1 of the iteration phase, the every term Δ_s^k is evaluated by a one-pass comparison against every $\Delta_C \in C, C \in F_i, F_i \in A$. Because $|\Delta_C| = 1$ in FACETS, the complexity is $O(n|\Delta||V|)$, assuming the worst case of $|V|$ clusters per facet. Step 2 of the iteration phase utilizes the OMEGADECOMPOSE procedure, which is based on the FUSE algorithm that takes $O(|\Delta|^2|V|^2)$ time in the worst case. Because the FUSE algorithm is executed for each facet, the complexity in this step is $O(n|\Delta|^2|V|^2)$. The total complexity per round is $O(n|\Delta||V| + n|\Delta|^2|V|^2)$, implying a polynomial time complexity at worst possible case.

3 SUPPLEMENTARY MATERIAL C: EVALUATION MEASURES

Given two decompositions (or facets) f_1 and f_2, the *Jaccard index* is defined as $J(f_1, f_2) = \frac{A}{A+B+C}$, where A is the number of protein pairs that is co-clustered in both f_1 and f_2, B is the number of protein pairs co-clustered in f_1 but not f_2, and C is the number of protein pairs co-clustered in f_2 but not f_1. $J(f_1, f_2)$ ranges from 0 to 1 (for identical clusterings).

4 SUPPLEMENTARY MATERIAL E: RUNNING TIME.

Figures 1(a)-(b) plot the running times of FACETS with varying network sizes $|V|$ and facet count n. Observe that the running time of FACETS on the largest network (human) is less than 3 minutes with $n = 11$ and less than a minute with $n = 2$.

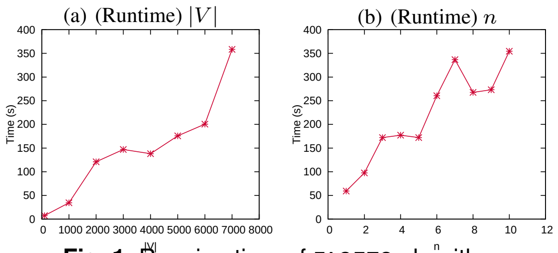

Fig. 1. Running time of FACETS algorithm.

REFERENCES

Agarwal, G. and Kempe, D. (2008) Modularity-maximizing graph communities via mathematical programming. *Eur. Phys. J. B*, 66(3).

Bader, G. and Hogue, C. (2003) An automated method for finding molecular complexes in large protein interaction networks. *BMC Bioinformatics*, 27.

Barabási, A.L. and Oltvai, Z.N. (2004) Network biology:understanding the cell's functional organization. *Nat. Rev. Genet.*, 5(2).

Ben-Hur, A. *et al.* (2002) A stability based method for discovering structure in clustered data. *In Pacific Symposium on Biocomputing*.

Botton, L. and Bengio, Y. (1994) Convergence properties of the k-means algorithms. *In Advances in Neural Information Processing Systems 7 (NIPS94).*

Brohée, S. and van Helden, J. (2006) Evaluation of clustering algorithms for protein-protein interaction networks. *BMC bioinformatics*, 7.

Caruana, R. *et al.* (2006) Meta Clustering. *In Proceedings of the Sixth IEEE International Conference on Data Mining*.

Chan, P.K *et al.* (1993) Spectral K-way ratio-cut partitioning and clustering. In DAC.

Cui,Y. *et al.* (2007) Non-Redundant Multi-View Clustering Via Orthogonalization. *In Proceedings of the Seventh IEEE International Conference on Data Mining*.

Daniel, J.A. *et al.* (2004) Deubiquitination of Histone H2B by a Yeast Acetyltransferase Complex Regulates Transcription. *J. Biol. Chem.*, 279, 1867-1871.

Fowlkes, E.B. and Mallows, C.B. (1983) A method for comparing two hierarchical clusterings. *Journal of the American Statistical Association*, 78(383).

Jagota, A. (1995) Approximating maximum clique with a Hopfield network. *IEEE Trans Neural Netw*, 6(3).

Kerrien, S. *et al.* (2007) IntAct-open source resource for molecular interaction data. *Nucleic Acids Res.*,35.

Koutelou, E. *et al.* (2010) Multiple faces of the SAGA complex. *Current Opinion in Cell Biology.* 22(3), 374382.

Krogan, N.J. *et al.* (2006) Global landscape of protein complexes in the yeast Saccharomyces cerevisiae. *Nature*, 440(7084).

Lavallée-Adam, M. *et al.* (2009) Detection of locally over-represented GO terms in protein-protein interaction networks. *In Proceedings of the 13th Annual International Conference on Research in Computational Molecular Biology*.

Massen, C.P. and Doye, J.P. (2005) Identifying communities within energy landscapes. *Phys. Rev. E*, 71(4).

Mewes, H.W. *et al.* (2002) MIPS: a database for genomes and protein sequences. *Nucleic Acids Res.*,30(1).

Navlakha, S. and Kingsford, C. (2010) Exploring biological network dynamics with ensembles of graph partitions. *In Pacific Symposium on Biocomputing*.

Niu, D. and Dy, J.G. (2010) Multiple Non-Redundant Spectral Clustering Views. *In ICML.*

Qi,Z. and Davidson, I. (2009) A principled and flexible framework for finding alternative clusterings. *In Proceeding of the 15th ACM SIGKDD international conference on Knowledge discovery in data mining*.

Rivera, C.G. *et al.* (2010) Network module identification in Cytoscape. *BMC bioinformatics*,11(1).

Seah,B.S *et al.* (2011) FUSE: Towards multi-level functional summarization of protein interaction networks. *In Proceedings of the 2nd ACM Conference on Bioinformatics, Computational Biology and Biomedicine (ACM BCB)* .

Wagstaff, K. and Cardie, C. (2000) Clustering with Instance-Level Constraints. *In Proceedings of the 17th international conference on Machine learning*.
